# Is the covering of the resection margin after distal pancreatectomy advantageous?

**DOI:** 10.1186/2047-783X-18-33

**Published:** 2013-09-28

**Authors:** Aycan Akca, Peter E Goretzki, Denis Wirowski, Marc A Renter, Edwin Bölke, Christiane Matuschek, Peter Arne Gerber, Bernhard J Lammers

**Affiliations:** 1 Department of Visceral and Endocrine Surgery, Lukaskrankenhaus Neuss, Preussenstr. 84, Neuss, 41464, Germany; 2 Medical Faculty, Department of Radiation Oncology, Heinrich Heine University Düsseldorf, Moorenstrasse 5, Düsseldorf, 40225, Germany; 3 Medical Faculty, Department of Dermatology, Heinrich Heine University Düsseldorf, Moorenstrasse 5, Düsseldorf, 40225, Germany

**Keywords:** Pancreas, Surgery, Resection margin, Distal pancreatectomy

## Abstract

**Background:**

In recent years, many advances in pancreatic surgery have been achieved. Nevertheless, the rate of pancreatic fistula following pancreatic tail resection does not differ between various techniques, still reaching up to 30% in prospective multicentric studies. Taking into account contradictory results concerning the usefulness of covering resection margins after distal pancreatectomy, we sought to perform a systematic, retrospective analysis of patients that underwent distal pancreatectomy at our center.

**Methods:**

We retrospectively analysed the data of 74 patients that underwent distal pancreatectomy between 2001 and 2011 at the community hospital in Neuss. Demographic factors, indications, postoperative complications, surgical or interventional revisions, and length of hospital stay were registered to compare the outcome of patients undergoing distal pancreatectomy with coverage of the resection margins vs. patients undergoing distal pancreatectomy without coverage of the resection margins. Differences between groups were calculated using Fisher’s exact and Mann–Whitney U test.

**Results:**

Main indications for pancreatic surgery were insulinoma (n=18, *24%*), ductal adenocarcinoma (n=9, *12%*), non-single-insulinoma-pancreatogenic-hypoglycemia-syndrome (NSIPHS) (n=8, *11%*), and pancreatic cysts with pancreatitis (n=8, *11%*). In 39 of 74 (*53%*) patients no postoperative complications were noted. In detail we found that 23/42 (*55%*) patients with coverage vs. 16/32 (*50%*) without coverage of the resection margins had no postoperative complications. The most common complications were pancreatic fistulas in eleven patients (*15%*), and postoperative bleeding in nine patients (*12%*). Pancreatic fistulas occurred in patients without coverage of the resection margins in 7/32 (*22%*) vs. 4/42 (10*11%*) with coverage are of the resection margins, yet without reaching statistical significance. Postoperative bleeding ensued with equal frequency in both groups (*12%* with coverage versus *13%* without coverage of the resection margins). The reoperation rate was *8%*. The hospital stay for patients without coverage was 13 days (5–60) vs. 17 days (8–60) for patients with coverage.

**Conclusions:**

The results show no significant difference in the fistula rate after covering of the resection margin after distal pancreatectomy, which contributes to the picture of an unsolved problem.

## Background

Recent advances in surgical techniques have helped to optimize the outcome of pancreatic surgery, reducing mortality rates to *3-5%*[1-4]. In the last 20 years several studies have focused on distal pancreatectomy, which accounts for up to *10%* of all pancreatic resections [5]. The main cause of postoperative morbidity in surgery of the distal pancreas is pancreatic fistulas (*30%*) [2,4,6,7]. Despite the advantages of laparoscopic distal pancreatectomy with reduction of postoperative pain, wound infection rates, and intraoperative blood loss, there are no significant differences in the rate of pancreatic fistulas when compared to open surgery [8-10]. In this context, many different surgical techniques for pancreatic resection and closure of the resection margin were described, such as closure by stapler with or without suture, pancreaticoenteric anastomoses with various modifications, mesh-application, or closure with fibrin glue. None of these techniques were superior concerning the rate of pancreatic fistulas [1,5,6,11].

The aim of this study was to evaluate, whether coverage of the resection margins after distal pancreatectomy reduced postoperative fistulas in our hands.

## Methods

We retrospectively analysed all patients receiving a distal pancreatic resection at our department between 2001 and 2011. We compared age, gender, diagnosis; postoperative complications, revision surgery and hospital stay in patients with coverage of the distal pancreatic margin (C) versus those without (NC). Ethical approval is not needed.

The indication for a covered stump was met by the surgeon - on personal discretion and if there was a soft pancreatic remnant.

In all cases – after mobilization and transection of the pancreatic tail – the resection margin was closed with monofil 4×0 PDS with separate sutures of the pancreatic duct. In some patients of the no cover-group, fibrin glue or a fibrinogen fleece was placed on the area of resection. For coverage of the resection margins, either the posterior gastric wall, or part of the omentum majus were fixed to the pancreatic tissue. In cases of gastric coverage, a small gastric serosal patch was performed. All patients received a drainage, which remained until suspension of high amylase fluid secretion. During laparoscopic surgery the remnant pancreas was closed with a stapler.

Pancreatic fistulas were primarily treated conservatively, either by leaving the intraoperatively drainage in place or by insertion of a new computed tomography (CT)-guided drainage. Pancreatic fistulas were defined using the classification of ISGPF, claiming a three-fold increase in amylase concentration of the drained secretion compared to the serum amylase at the third postoperative day. Clinically, three levels of pancreatic fistulas, A, B and C, were differentiated as defined by Bassi et al. [12].

Descriptive statistics were used to analyse the variables: differences between groups were statistically evaluated using either Fisher’s exact test (categorical variables) or Mann–Whitney U test (metric variables). *p* values of less than *0.05* were considered as statistically significant.

## Results

Between 2001 and 2011, 74 patients underwent a distal pancreatectomy with (n=42) or without (n=32) coverage of the resection margin. In 42 of 74 patients (*57%*) the resection margin was closed either by patching it to the gastric wall (n=35, *83%*), or by attaching part of the omentum majus (n=7, *17%*) (C). In 32 of 74 patients (*43%*) there was no coverage of the resection margin (NC) besides direct suture or fibrin glue, as described above. The median age was 53 years in the cover-group (range 25–88 years) and 54 years in no cover-group (range 30–80 years), respectively. 45 of 74 patients (61%) were female. These parameters were not significantly different between the cover- and no cover-group (see Table 1).

**Table 1:** Demographic and perioperative data

	**Without cover**	**With gastric cover**	**With omentum majus**	
	**n=32**	**N=35**	**n=7**	
**Age (years)**	54 (30–80)	53 (25–82)	51 (29–88)	*n.s.*^*1*^
**Gender**				*n.s.*^*2*^
**Female**	18	21	6	
**Male**	14	14	1	
**Laparoscopic**	10, 2 conv.	2	0	
**Hospital stay (days, median, range 6–60)**	13 (6–60)	17 (8–60)	15 (10–24)	*n.s.*^*1*^

Demographic and perioperative data of 74 patients with and without covering. Results were not significantly different using Mann–Whitney and Fisher’s exact test.

^1^Mann-Whitney U test, ^2^Fisher’s exact test; conv: conversion to open resection.

n.s.: not significant.

The most common indications for surgery were insulinomas (18/74, *24%*), followed by ductal adenocarcinomas of the pancreatic tail (9/74, *12%*), non-single-insulinoma-pancreatogenic-hypoglycemia-syndrome (NSIPHS) (8/74, *11%*), pancreatic cysts (8/74, *11%*), multiple endocrine neoplasia I (MEN I) (7/74, *9%*), and for five patients each (*7%*) pancreatic neuroendocrine neoplasias (pNEN) or intrapancreatic metastasis. Indications were equally distributed between both groups (see Table 2).

**Table 2:** Indications for surgery

	**Total**	**Without cover**	**With gastric cover**	**With omentum majus**
	**n=74**	**n=32**	**n=35**	**n=7**
Insulinoma	18	9	6	3
Ductal adeno-CA	9	3 (R0)	5 (n=1 R1)	1 (R0)
NIPHS	8	4	4	
Cyst	8	2	4	2
MEN I	7	4	3	
NET	5	3	1	1
Metastasis	5	1	4	
Chronic pancreatitis	4	1	3	
Malign. Insulinoma	3	2	1	
Gastronoma	2	1	1	
Acute biliary pancreatitis	1		1	
MEN IIa	1		1	
Serotoninoma	1	1		
Mucinous tumour	1		1	
Accessory spleen in the pancreatic tail	1	1		

The most common indications for surgery were insulinomas (18/74, 24%).

*CA*, carcinoma.

*MEN*, multiple endocrine neoplasia.

*NET*, neuroendocrine tumour.

*NIPHS*, non-insulinoma-pancreatogen-hypoglycamia-syndroma.

In twelve patients (*16%*) the distal pancreatectomy was performed laparoscopically, in two patients with and in ten patients without coverage of the resection margins. In two cases of this group the laparoscopic procedure had to be converted to an open access: in one patient the preoperative described insulinoma was not detected by laparoscopy, and in the other case the soft resection margin was estimated insufficiently closed, leading to conversion to open resection and coverage (see Table 1).

R0-resection was achieved in eight of nine patients (*89%*) with a ductal adenocarcinoma of the pancreatic tail. Only a 77-year-old male patient had a R1 resection because of vascular invasion by the tumour.

In 39 of 74 patients (*53%*) we observed no postoperative complications, with 23 patients (*55%*) in the cover-group and 16 patients (*50%*) in the no cover-group (n.s.) (see Table 3). The most frequent complications were pancreatic fistulas in eleven patients (*15%*), bleeding in nine patients (*12%*), of which four (*44%*) needed operative revision, and pneumonia in eight patients (*11%*). Patients of the no cover-group showed a higher frequency of pancreatic fistulas as compared to those of the cover-group (7/32 (*22%*) versus 4/42 (*10%*) (n.s.). Seven of eleven patients with a pancreatic fistula were asymptomatic, just detecting high amylase levels in the drainage (ISGPF A). Three of eleven patients suffered from a pancreatic fistula ISGPF B, and one patient with a persistent pancreatic fistula (ISGPF C) had to be reoperated. Postoperative bleeding occurred in both groups with similar frequency (5/42, *12%* versus 4/32, *13%*) (n.s.).

**Table 3:** Complications

	**Without cover**	**With gastric cover**	**With omentum majus**	**Total**	
	**n=32**	**n=35**	**n=7**	**n=74**	
No complications	16 (50%)	17 (49%)	6 (86%)	39	*n.s.*^*2*^
Complications	16 (50%)	18 (51%)	1	35	*n.s.*^*2*^
Fistula	7(22%)	4 (11%)		11	*n.s.*^*2*^
ISGPF A	5	2			
ISGPF B	2	1			
ISGPF C	0	1			
Bleeding	4	4	1	9	*n.s.*^*2*^
Pneumonia	1	7		8	*n.s.*^*2*^
Pancreatitis	0	3		3	*n.s.*^*2*^
Pseudocyst	1	2		3	*n.s.*^*2*^
Splenic infarction	2			2	*n.s.*^*2*^
Abscess		1		1	*n.s.*^*2*^
Small bowel fistula	1			1	*n.s.*^*2*^
Perforation of the colon sigmoideum	1			1	*n.s.*^*2*^

The most common complications were pancreatic fistulas in 11 patients (15%), and 9 patients (12%) developed postoperative bleeding. Statistical differences between the groups were not significant using Fisher’s exact test.

ISGPF: Postoperative pancreatic fistula: an international study group (ISGPF) definition.

^2^Fisher’s exact test; n.s.: not significant.

Rarer complications included pancreatitis or pancreatic pseudocysts (three patients each), splenic infarction (two cases), pancreatic abscess, small bowel fistula, and iatrogenic perforation of the colon sigmoideum (one case each) (see Table 3).

No patient died within the first 30 days after surgery. One patient died due to sepsis following multiple organ failure at day 72 after surgery. This patient with a malignant serotoninoma of the pancreas had liver metastases (TNM pT2 pN1 M1 V1 G2) and a complicated postoperative course with a small bowel fistula, severe pneumonia, and splenic infarction two months after initial surgery.

Six of 74 (*8%*) patients had to undergo revision surgery. Main indication for revision was postoperative bleeding [two patients in each group (*5%*)]. Reasons for postoperative bleeding were a diffuse bleeding in the area of resection margin (n=2), a factor XIII deficiency (n=1), and a diffuse bleeding in the area of the ligamentum falciforme (n=1), respectively.

One patient without coverage had to be reoperated on due to a small bowel fistula. In one patient with a gastrinoma and neuroendocrine tumours of the pancreas in MEN I, resurgery was neccessary two months after distal pancreatectomy because of a persistent fistula despite conservative treatment.

Pseudocysts occurred in three patients, with no revision necessary.

The median hospital stay was 16.5 days for all patients (range 6–60) and was shorter in patients in the no cover-group (13 days) as compared to those in the cover-group (17 days) (see Table 1). This was not significant.

The outcome of our retrospective analysis showed no significant difference in the fistula rate after covering of the resection margin after distal pancreatectomy.

## Discussion

Nowadays in experienced hands pancreatic resection has a low mortality rate of less than *5%*[1-4]. In the current literature, a morbidity rate of *30-50%* is reported, with pancreatic fistulas followed by bleeding and infection as the main causes [2,4-7].

Also in our patients, pancreatic fistula was the most common postoperative complication. Nevertheless, our study reported a rather low rate of pancreatic fistulas (*15%*) as compared to the experiences of other groups (*12-30%*) (see Table 4) [2-4,6,8-10,13-15]. Moreover, our analysis revealed a lower frequency of post-operative fistulas in patients undergoing coverage of resection margins (C, 10*11%)* as compared to patients undergoing surgery without coverage (NC, *22%)*, yet without reaching statistical significance (see Table 3). Notably, all except one patient with a persistent pancreatic fistula could be treated conservatively. Since the drain management and removal was not defined in this study, the overall and detailed fistula rate might be biased.

**Table 4:** Rates of pancreatic fistula (in %) after distal pancreatectomy

**Author**	**Year**	**Group size**	**Procedures**					
			**Open without cover**			**Open with cover**	**Lap without cover**	**Lap with cover**
			**Stapler**	**Suture**	**Others**			
Present study	2012	74		22		11	17	0
Limongelli	2012	52			20 (comb)		18	
Hackert	2011	98		13	21 (enucleation)			
Diener	2011	352 (21c)	32	28				
Song	2011	359					38	4
Kim	2008	128		14			9	
Pugliese	2008	14					29	0
Kleef	2007	302	16	9		8		
Bilimoria	2003	126	20	22	10 (PDL)			
					34 (without PDL)			
Adam	2001	41		29		7		
Suzuki	1999	58			4 (UD+ PDL)			
					26 (CV)			

Table four compares the rates of pancreatic fistula (in %) after distal pancreatectomy with other works.

c: centers; comb: combined techniques (ultrasonic dissection, sutures, staples, sutures and combinations); *PDL*, pancreatic duct ligation; *UD*, ultrasonic dissection; *CV*, conventional.

Furthermore we did not define or rule out the perioperative use of octreotide, which could be another form of potential bias in this retrospective analysis.

Due to the limited number of patients included in our analysis conclusions about the impact of diagnosis, comorbidities, or over-all patient outcomes were limited and not drawn.

In recent years also other groups focussed on the effect of coverage of the pancreatic stump on the frequency of postoperative pancreatic fistula, yet also without gaining clear results. In our opinion, the influence of personal experience and skill seems to surpass the impact of various technical approaches.

Different other surgical techniques have been described to minimize the rate of postoperative pancreatic fistulas, which varies between *7%* and *33%*[2-4,8,11]. Besides the limitation that due to the inhomogeneity of patient groups and procedures being compared a assessment of published studies on this topic is difficult but it seems that no method could demonstrate clear superiority to others.

While Kleef et al. described an increased incidence of pancreatic fistulas after stapler closure (*15.9%* in the stapler group versus *9.3%* in the suture group versus *0%* in the pancreaticojejunostomy group), other studies could not verify this finding (see Table 4) [1,2,6,11,13,14]. Down this line no difference in the rate of pancreatic fistulas when comparing suture and stapler method or a combination of both could be proven [1,5,6].

Laparoscopic distal pancreatectomy failed to reduce the rate of pancreatic fistulas significantly, with rates of *9-18%* versus *14-20%* for open procedures [8-10,16-19]. This corresponds to our own results, with a rate of *17%* for laparoscopic resection versus *22%* for open distal pancreatectomy.

Other groups showed reduced rates of pancreatic fistulas by varying the operative technique, but data were not generated by prospective randomized trials. In these prospective and retrospective analyses stapler-closure, placement of a drainage and covering of the pancreatic stump by stomach and fibrin reduced the rate of pancreatic fistulas from *38%* to *4%*[9]. These findings are in line with our experiences, demonstrating that coverage of the pancreatic margin resulted in a tendency toward a reduced rate of pancreatic fistulas (10*11%* versus *22%*). The selective ligation of the pancreatic duct was shown to reduce the rate of pancreatic fistulas to less than *5-10%*[14,15]. Yet, neither of the discussed studies nor our study presented here fulfils the requirements of prospective randomised trials.

Another important point was that the overall fistula rate was 15%, which clearly differs from the overall fistula rate of randomized multicentre trials. It might be that this significantly lower fistula rate is due to the small sample size of this series, biased outcome assessment as outlined above or due to a special, superior surgical technique and perioperative care. This significant discrepancy leads to an impaired comparability with current randomized collectives, which impairs the interpretative power of these results.

## Conclusion

Additional covering of the pancreatic stump using sutures does not add any benefit and, therefore, will not be required.

## Competing interests

The authors declared that they have no competing interests.

## Authors’ contributions

All authors participated in the publication preparation and drafted the manuscript. AA collected all clinical data. All authors read and approved the final manuscript.
